# Data on gray matter alterations in anxious depression

**DOI:** 10.1016/j.dib.2019.104322

**Published:** 2019-07-30

**Authors:** Wei Peng, Zhiyun Jia, Xiaoqi Huang, Su Lui, Weihong Kuang, John A. Sweeney, Qiyong Gong

**Affiliations:** a Huaxi MR Research Center (HMRRC), Department of Radiology, West China Hospital of Sichuan University, Chengdu, China; b Department of Radiology, People's Hospital of Deyang City, Deyang, China; c Department of Nuclear Medicine, West China Hospital of Sichuan University, Chengdu, China; d Department of Psychiatry, West China Hospital of Sichuan University, Chengdu, China; e Department of Psychiatry and Behavioral Neuroscience, University of Cincinnati, OH, USA; f Research Unit of Psychoradiology (2018RU011), Chinese Academy of Medical Sciences, Sichuan University, Chengdu 610041, China

**Keywords:** Anxious depression, Gray matter, Non-anxious depression

## Abstract

This data article provided additional data related to the research article entitled “Brain structural abnormalities in emotional regulation and sensory processing regions associated with anxious depression” Peng et al.,2019. Correlation analyses were conducted for clinical information (HAMD total, anxiety/somatization scores, HAMA total and illness duration) and identified regional gray matter volumes in all patients with anxious depression and non-anxious depression. More detailed correlation analysis was applied for each item of anxiety factor and reginal gray matter volumes to find which items were more associated with structural alterations in patients. Data showed that mean values of regional gray matter volumes in left postcentral gyrus (PCG) were positively associated with HAMD total and anxiety factor scores in anxious depression group. More detailed correlation analysis considering each item of anxiety factor revealed that, item 10 (psychic anxiety) and Item15 (hypochondriasis) were most significantly and positively associated with regional gray matter volumes in left PCG in anxious group. While HAMA scores and illness duration showed no significant correlation with any regional gray matter volume in both patient groups. Sample size matched groups were selected to explore possible replicability of imaging results. It revealed that different gray matter volumes in right inferior frontal gyrus were most robust findings among three groups. And anxious group had larger gray matter volumes in left PCG than non-anxious depression, despite of not survived after multiple comparisons corrections.

**Table:** Specifications Table

Subject area	Psychiatry
More specific subject area	Major depressive disorder
Type of data	Table
How data was acquired	Evaluation from psychiatric doctors when interviewing the patients,3DT1-weighted images of all participants.
Data format	Raw and Analyzed
Experimental factors	3DT1-weighted images, HAMD scores, anxiety factor scores, HAMA scores, illness duration, identified gray matter volumes
Experimental features	Voxel-based morphometry analysis, Correlation analyses, Multiple comparison corrections
Data source location	West China Hospital of Sichuan University, Chengdu, China
Data accessibility	Data is with this article
Related research article	Peng et al. “Brain structural abnormalities in emotional regulation and sensory processing regions associated with anxious depression”, *Prog Neuropsychopharmacol Biol Psychiatry*, 94 (2019):109676. https://doi.org/10.1016/j.pnpbp.2019.109676.

**Table:** 

**Value of the data** • Reflecting the possible association between clinical information and neuroimaging parameters. • Providing new insight for future experiments on anxious depression. • May serve as region-of-interest for other neuroimaging studies on anxious depression.

## 1 Data

This Data in Brief provided detailed results of correlation analyses between clinical characteristics (HAMD total, anxiety/somatization score, HAMA total and illness duration) and extracted mean values of regional gray matter volumes in whole anxious depression and non-anxious depression groups (Tables 1–5) [1]. The raw individual values of correlation analyses for each patient were listed in Tables 8–9 In addition, this data article presented imaging results for matched groups (Tables 6–7) [1].

## 2 Experimental design, materials and methods

### 2.1 Correlation analyses between clinical information and identified regional gray matter volumes in whole groups

Pearson correlation analysis was conducted for symptom severity (HAMD total, anxiety/somatization scores and HAMA total) and extracted gray matter volumes in whole patients groups. More detailed correlation analysis was applied for each item of anxiety factor and reginal gray matter volumes to find which items were more associated with structural alterations in patients. Nonparametric Spearman correlation analysis was conducted for illness duration and the extracted gray matter volumes, as the distribution of illness duration was non-normal. Multiple comparisons were controlled by Bonferroni correction using p < 0.05/n (n: number of the identified clusters) to avoid spurious weak relationships [2].

### 2.2 Voxel based morphometry analysis of matched groups

To explore the possible replicability of the results, we further investigated the imaging findings of sample size matched groups (n = 50 for each group) with age and gender maximally matched. The imaging analysis procedure for matched groups was conducted under standard Diffeomorphic Anatomical Registration using the Exponentiated Lie algebra (DARTEL) process implemented in Statistical Parametric Mapping (SPM) software running under Matlab 7.10 (Math Works, Natick, MA, USA) [3]. The preprocessing procedure was as follows: setting the image origin to the anterior commissure; applying the DARTEL toolbox with standard setting for all steps to generate a high-dimensional normalization protocol used to segment gray matter. Individual flow-fields resulting from the DARTEL registration to the reference template were used to warp the gray matter segments. The warped gray matter maps for each subject were modulated with the Jacobian determinant from the DARTEL registration fields to preserve tissue amounts, in which each voxel in the resulting images had an absolute amount of brain volume corresponding to the brain volume per unit. Finally, modulated warped gray matter segments were smoothed with a Gaussian smoothing kernel of 8 mm full-width at half maximum (FWHM). All preprocessed gray matter maps were examined by visual inspection for overall segmentation and registration accuracy.

After these preprocessing steps, the resulting gray matter maps for the three groups were compared by analysis of covariance (ANCOVA). Multiple comparison corrections were applied through Monte Carlo simulations in AlphaSim program of the Resting-State fMRI Data Analysis Toolkit (REST), with individual voxel p = 0.005, 1,000 simulations, and FWHM = 6 mm, to reach corrected significance level of p < 0.05 [4].
