# Supplementary material for: Data on gray matter alterations in anxious depression
Source: Data Brief. 2019 Jul 30;25:104322. doi: 10.1016/j.dib.2019.104322 (PMC6702383; doi:10.1016/j.dib.2019.104322)
Supplement: Supplementary file 1 [file mmc1.doc]

**Table 1.** Correlation analysis between HAMD scores and mean gray matter volumes in identified clusters in whole patients groups

| Identified Clusters | MDD+A | | | | MDD-A | | | |
| --- | --- | --- | --- | --- | --- | --- | --- | --- |
|  | Pearson **r** | 95% CI | p | Corrected significance* | Pearson **r** | 95% CI | p | Corrected significance* |
| R IFG | -0.096 | -0.284 to 0.098 | 0.332 | No | 0.124 | -0.141 to 0.373 | 0.358 | No |
| R OFG | -0.123 | -0.309 to 0.071 | 0.212 | No | 0.168 | -0.097 to 0.410 | 0.213 | No |
| Bilateral Culmen | 0.024 | -0.169 to 0.216 | 0.805 | No | 0.155 | -0.110 to 0.399 | 0.250 | No |
| L PCG | 0.273 | 0.084 to 0.442 | 0.005 | Yes | 0.173 | -0.092 to 0.415 | 0.198 | No |
| L Cuneus | 0.031 | -0.162 to 0.223 | 0.752 | No | 0.131 | -0.134 to 0.379 | 0.330 | No |

*Significant level was set as p < 0.01 (0.05/5) for corrections of multiple comparisons. Positive association was observed only between gray matter volumes in the L PCG and HAMD scores in MDD+A group (r = 0.273, p = 0.005).

Abbreviations: CI, confidence intervals; HAMD, Hamilton Depression Scale; IFG, inferior frontal gyrus; L, left; MDD+A, anxious depression; MDD-A, non-anxious depression; OFG, orbital frontal gyrus; PCG, postcentral gyrus; R, right.

**Table 2.** Correlation analysis between anxiety/somatization scores and mean gray matter volumes in identified clusters in whole patients groups

| Identified Clusters | MDD+A | | | | MDD-A | | | |
| --- | --- | --- | --- | --- | --- | --- | --- | --- |
|  | Pearson **r** | 95% CI | p | Corrected significance* | Pearson **r** | 95% CI | p | Corrected significance* |
| R IFG | -0.014 | -0.206 to 0.179 | 0.885 | No | -0.094 | -0.346 to 0.171 | 0.486 | No |
| R OFG | 0.024 | -0.169 to 0.216 | 0.805 | No | -0.061 | -0.316 to 0.203 | 0.653 | No |
| Bilateral Culmen | 0.033 | -0.161 to 0.224 | 0.741 | No | -0.042 | -0.300 to 0.221 | 0.755 | No |
| L PCG | 0.289 | 0.102 to 0.456 | 0.003 | Yes | 0.119 | -0.146 to 0.369 | 0.377 | No |
| L Cuneus | 0.042 | -0.152 to 0.233 | 0.671 | No | -0.024 | -0.283 to 0.238 | 0.861 | No |

*Significant level was set as p < 0.01 (0.05/5) for corrections of multiple comparisons. Positive association was observed only between gray matter volumes in the L PCG and anxiety/somatization scores in MDD+A group (r = 0.289, p = 0.003).

Abbreviations: CI, confidence intervals; HAMD, Hamilton Depression Scale; IFG, inferior frontal gyrus; L, left; MDD+A, anxious depression; MDD-A, non-anxious depression; OFG, orbital frontal gyrus; PCG, postcentral gyrus; R, right.

**Table 3.** Correlation analysis between illness durations and mean gray matter volumes in identified clusters in whole patients groups

| Identified Clusters | MDD+A | | | | MDD-A | | | | | |
| --- | --- | --- | --- | --- | --- | --- | --- | --- | --- | --- |
|  | Spearman **rho** | 95% CI | p | Corrected significance* | | Spearman **rho** | 95% CI | p | Corrected significance* | |
| R IFG | 0.112 | -0.089 to 0.303 | 0.259 | No | 0.060 | | -0.211 to 0.323 | 0.657 | | No |
| R OFG | 0.236 | 0.039 to 0.414 | 0.016 | No | 0.118 | | -0.155 to 0.374 | 0.381 | | No |
| Bilateral Culmen | -0.004 | -0.202 to 0.194 | 0.968 | No | -0.011 | | -0.278 to 0.258 | 0.938 | | No |
| L PCG | -0.057 | -0.253 to 0.142 | 0.562 | No | 0.175 | | -0.098 to 0.423 | 0.194 | | No |
| L Cuneus | 0.031 | -0.168 to 0.228 | 0.754 | No | 0.094 | | -0.178 to 0.354 | 0.485 | | No |

* Significant level was set as p < 0.01 (0.05/5) for corrections of multiple comparisons.

Abbreviations: CI, confidence intervals; HAMD, Hamilton Depression Scale; IFG, inferior frontal gyrus; L, left; MDD+A, anxious depression; MDD-A, non-anxious depression; OFG, orbital frontal gyrus; PCG, postcentral gyrus; R, right.

**Table 4.** Correlation analysis between each item of anxiety factor, HAMA scores and mean gray matter volumes in identified clusters in MDD+A groups

| Region | Item 10 | Item 11 | Item 12 | Item 13 | Item 15 | Item 17 | HAMA |
| --- | --- | --- | --- | --- | --- | --- | --- |
|  | p r | p r | p r | p r | p r | p r | p r |
| R IFG | 0.258 -0.112 | 0.768 0.029 | 0.659 -0.044 | 0.756 -0.031 | 0.964 0.004 | 0.117 0154 | 0.816 0.023 |
| R OFG | 0.705 -0.038 | 0.608 0.051 | 0.024 -0.221 | 0.286 -0.106 | 0.085 0.170 | 0.234 0.118 | 0.831 -0.021 |
| Bilateral Culmen | 0.153 -0.141 | 0.832 -0.021 | 0.747 0.032 | 0.266 -0.110 | 0.266 0.110 | 0.010 0.252 | 0.923 0.010 |
| L PCG | 0.001* 0.323 | 0.240 0.116 | 0.552 -0.059 | 0.976 0.003 | 0.004* 0.282 | 0.141 -0.145 | 0.231 0.118 |
| L Cuneus | 0.416 -0.081 | 0.934 0.008 | 0.253 -0.113 | 0.178 -0.133 | 0.015 0.237 | 0.291 0.103 | 0.195 0.128 |

* Significant level was set as p < 0.01 (0.05/5) for corrections of multiple comparisons.

Abbreviations: HAMA, Hamilton Anxiety Scale; IFG, inferior frontal gyrus; L, left; MDD+A, anxious depression; OFG, orbital frontal gyrus; PCG, postcentral gyrus; R, right.

**Table 5.** Correlation analysis between each item of anxiety factor, HAMA scores and mean gray matter volumes in identified clusters in MDD-A groups

| Region | Item 10 | Item 11 | Item 12 | Item 13 | Item 15 | Item 17 | HAMA |
| --- | --- | --- | --- | --- | --- | --- | --- |
|  | p r | p r | p r | p r | p r | p r | p r |
| R IFG | 0.871 -0.022 | 0.968 0.005 | 0.801 -0.034 | 0.127 -0.205 | 0.118 0.209 | 0.115 -0.211 | 0.348 -0.127 |
| R OFG | 0.251 -0.155 | 0.985 0.003 | 0.426 -0.107 | 0.538 0.083 | 0.030 0.288 | 0.078 -0.236 | 0.716 -0.049 |
| Bilateral Culmen | 0.893 0.018 | 0.906 0.016 | 0.192 -0.175 | 0.891 -0.018 | 0.292 0.142 | 0.270 -0.149 | 0.181 -0.180 |
| L PCG | 0.842 -0.027 | 0.274 0.147 | 0.795 0.035 | 0.868 0.023 | 0.088 0.228 | 0.111 -0.213 | 0.988 -0.002 |
| L Cuneus | 0.480 -0.095 | 0.705 -0.051 | 0.899 0.017 | 0.906 0.016 | 0.355 0.125 | 0.913 -0.015 | 0.732 -0.046 |

* Significant level was set as p < 0.01 (0.05/5) for corrections of multiple comparisons.

Abbreviations: HAMA, Hamilton Anxiety Scale; IFG, inferior frontal gyrus; L, left; MDD-A, non-anxious depression; OFG, orbital frontal gyrus; PCG, postcentral gyrus; R, right.

**Table 6.** Demographic and clinical characteristics of patients and healthy controls in matched groups

| Characteristic | MDD+A | | MDD-A | | HC | |  |
| --- | --- | --- | --- | --- | --- | --- | --- |
|  | Mean | SD | Mean | SD | Mean | SD | p value |
| Age, years | 30 | 9 | 30 | 9 | 30 | 9 | 0.971 |
| Illness duration, months | 5 | 5 | 4 | 10 |  |  | 0.609 |
| HAMD score | 28 | 6 | 24 | 6 |  |  | 0.001 |
| HAMA score | 29 | 8 | 17 | 5 |  |  | <0.001 |
| Anxiety/Somatization score | 9 | 2 | 5 | 1 |  |  | < 0.001 |
| Gender(Female/Male) | 32 | 18 | 32 | 18 | 32 | 18 | >0.999 |

Statistical significances between matched patient groups were observed for HAMD, Anxiety/Somatization and HAMA scores (p < 0.05). No other significant differences were observed between patient groups or among all groups.

Abbreviations: HAMA, Hamilton Anxiety Scale; HAMD, Hamilton Depression Scale; HC, healthy controls; SD, standard deviation; MDD+A, anxious depression; MDD-A, non-anxious depression.

**Table 7.** Differences in the gray matter volumes among patients with anxious depression, non-anxious depression, and healthy controls in sample size matched groups

| Anatomical region | MNI Coordinate | | | cluster size | F^a^ | Comparison (t)^b^ | | |
| --- | --- | --- | --- | --- | --- | --- | --- | --- |
|  | x | y | z |  |  | MDD+A vs. HC | MDD+A vs. MDD-A | MDD-A vs. HC |
| R IFG  L PCG | 52  -25 | 11  -45 | 22  57 | 2118  2138 | 10.83  -- | -4.15 | -3.67  3.97 |  |

^a^ Results survived a voxel-wise statistical threshold (p < 0.05), as corrected for multiple comparisons using AlphaSim program.

^b^ Voxel-wise t values for post hoc between-group comparisons.

Abbreviations: HC, healthy controls; IFG, inferior frontal gyrus; L, left; MDD+A, anxious depression; MDD-A, non-anxious depression; MNI, Montreal Neurological Institute; PCG, postcentral gyrus; R, right.

**Table 8**.Clinical information and mean gray matter volumes of identified clusters in each MDD+A patient

| R IFG | R OFG | Bilateral Culmen | L PCG | L Cuneus | HAMD | Anxiety scores | HAMA | Illness duration |
| --- | --- | --- | --- | --- | --- | --- | --- | --- |
| 0.496555 | 0.278032 | 0.420494 | 0.328519 | 0.513173 | 29 | 11 | 36 | 3.33 |
| 0.484538 | 0.263729 | 0.431751 | 0.502248 | 0.45958 | 30 | 11 | 33 | 25.00 |
| 0.589559 | 0.285351 | 0.410355 | 0.37876 | 0.519481 | 24 | 11 | 35 | 3.33 |
| 0.507312 | 0.258554 | 0.43972 | 0.35557 | 0.574258 | 28 | 10 | 26 | 1.00 |
| 0.473604 | 0.317184 | 0.429363 | 0.383651 | 0.456457 | 36 | 9 | 41 | 6.00 |
| 0.436337 | 0.24987 | 0.390938 | 0.508601 | 0.448338 | 35 | 10 | 43 | 2.33 |
| 0.479599 | 0.219986 | 0.349269 | 0.355332 | 0.436971 | 24 | 8 | 31 | 0.50 |
| 0.34132 | 0.198521 | 0.375361 | 0.379303 | 0.40497 | 32 | 9 | 36 | 3.33 |
| 0.432715 | 0.230295 | 0.377474 | 0.357073 | 0.475715 | 23 | 10 | 32 | 6.33 |
| 0.552211 | 0.306594 | 0.379259 | 0.239531 | 0.373946 | 26 | 10 | 33 | 73.00 |
| 0.32921 | 0.262827 | 0.321272 | 0.32752 | 0.349712 | 21 | 8 | 10 | 3.00 |
| 0.428963 | 0.256797 | 0.419811 | 0.433723 | 0.487189 | 33 | 13 | 33 | 3.33 |
| 0.558366 | 0.290043 | 0.431581 | 0.310156 | 0.538101 | 30 | 8 | 40 | 12.67 |
| 0.454638 | 0.331956 | 0.475589 | 0.431595 | 0.428905 | 23 | 10 | 25 | 25.00 |
| 0.476321 | 0.286676 | 0.524517 | 0.530729 | 0.484251 | 22 | 10 | 35 | 2.33 |
| 0.498386 | 0.251434 | 0.430759 | 0.521893 | 0.42265 | 39 | 14 | 36 | 25.00 |
| 0.360639 | 0.272229 | 0.411058 | 0.414389 | 0.338463 | 32 | 11 | 31 | 0.70 |
| 0.438879 | 0.267947 | 0.419563 | 0.337414 | 0.555827 | 30 | 10 | 35 | 0.47 |
| 0.394404 | 0.310812 | 0.46306 | 0.509459 | 0.428581 | 36 | 11 | 26 | 6.00 |
| 0.499286 | 0.289541 | 0.40947 | 0.511693 | 0.475392 | 25 | 10 | 19 | 6.00 |
| 0.399558 | 0.220961 | 0.365804 | 0.422736 | 0.375708 | 32 | 10 | 24 | 4.00 |
| 0.484181 | 0.251909 | 0.431071 | 0.40106 | 0.507591 | 39 | 12 | 33 | 6.33 |
| 0.393629 | 0.250047 | 0.394513 | 0.400985 | 0.445558 | 28 | 10 | 26 | 2.00 |
| 0.416598 | 0.23911 | 0.380809 | 0.446006 | 0.425616 | 23 | 8 | 25 | 6.33 |
| 0.414379 | 0.228252 | 0.35758 | 0.368579 | 0.439504 | 32 | 11 | 35 | 0.47 |
| 0.417058 | 0.250849 | 0.443412 | 0.355957 | 0.428936 | 20 | 9 | 13 | 6.33 |
| 0.388048 | 0.238926 | 0.368255 | 0.472983 | 0.453387 | 31 | 9 | 28 | 5.67 |
| 0.522233 | 0.282461 | 0.429679 | 0.451768 | 0.416742 | 27 | 10 | 40 | 6.60 |
| 0.456743 | 0.278716 | 0.41266 | 0.384154 | 0.350319 | 32 | 11 | 35 | 1.33 |
| 0.454389 | 0.233892 | 0.415726 | 0.42425 | 0.500839 | 27 | 10 | 32 | 0.50 |
| 0.465836 | 0.313652 | 0.46873 | 0.358317 | 0.497486 | 20 | 9 | 18 | 12.17 |
| 0.508984 | 0.251494 | 0.384993 | 0.387561 | 0.413578 | 34 | 11 | 37 | 5.33 |
| 0.482148 | 0.244699 | 0.334632 | 0.377962 | 0.368857 | 29 | 11 | 32 | 6.33 |
| 0.375139 | 0.228948 | 0.367341 | 0.37814 | 0.357733 | 28 | 11 | 29 | 1.33 |
| 0.41555 | 0.223109 | 0.328406 | 0.354988 | 0.476311 | 31 | 10 | 36 | 12.17 |
| 0.584109 | 0.273076 | 0.443141 | 0.365379 | 0.574977 | 18 | 9 | 24 | 5.13 |
| 0.359262 | 0.237711 | 0.34634 | 0.328058 | 0.439748 | 28 | 9 | 36 | 8.33 |
| 0.399174 | 0.235402 | 0.358519 | 0.439404 | 0.378111 | 22 | 8 | 19 | 2.00 |
| 0.452063 | 0.246605 | 0.402359 | 0.336581 | 0.415008 | 27 | 8 | 27 | 2.33 |
| 0.584842 | 0.276067 | 0.428842 | 0.389838 | 0.524583 | 25 | 11 | 34 | 0.50 |
| 0.429186 | 0.259723 | 0.424341 | 0.388149 | 0.533191 | 28 | 9 | 24 | 24.33 |
| 0.458479 | 0.249375 | 0.402838 | 0.385259 | 0.445605 | 25 | 8 | 30 | 1.33 |
| 0.441508 | 0.284061 | 0.414838 | 0.376666 | 0.466373 | 27 | 9 | 21 | 12.33 |
| 0.478731 | 0.244471 | 0.378348 | 0.310719 | 0.356245 | 29 | 9 | 34 | 12.67 |
| 0.518927 | 0.262198 | 0.371325 | 0.404678 | 0.56139 | 31 | 13 | 33 | 6.00 |
| 0.426196 | 0.240312 | 0.400939 | 0.399582 | 0.426323 | 26 | 9 | 25 | 6.33 |
| 0.439611 | 0.24902 | 0.455973 | 0.426745 | 0.433178 | 43 | 13 | 42 | 3.33 |
| 0.400512 | 0.275744 | 0.398368 | 0.40388 | 0.454022 | 22 | 8 | 22 | 4.33 |
| 0.438263 | 0.227446 | 0.364156 | 0.490579 | 0.529591 | 30 | 9 | 24 | 6.33 |
| 0.360639 | 0.165359 | 0.36295 | 0.401683 | 0.399706 | 31 | 8 | 35 | 2.00 |
| 0.456553 | 0.2335 | 0.461842 | 0.330049 | 0.383539 | 20 | 8 | 20 | 0.70 |
| 0.607361 | 0.286419 | 0.512608 | 0.327157 | 0.578489 | 31 | 8 | 34 | 12.17 |
| 0.490596 | 0.234924 | 0.38123 | 0.396148 | 0.508931 | 29 | 9 | 17 | 4.33 |
| 0.418341 | 0.247504 | 0.426106 | 0.561029 | 0.459153 | 30 | 10 | 29 | 1.33 |
| 0.432024 | 0.283845 | 0.443052 | 0.423041 | 0.394207 | 34 | 12 | 38 | 1.33 |
| 0.437254 | 0.215141 | 0.351507 | 0.356876 | 0.450023 | 25 | 8 | 31 | 12.67 |
| 0.508978 | 0.254871 | 0.412817 | 0.413252 | 0.457952 | 31 | 10 | 41 | 1.17 |
| 0.463805 | 0.293013 | 0.416293 | 0.310208 | 0.407675 | 22 | 8 | 27 | 1.33 |
| 0.341735 | 0.223044 | 0.328465 | 0.364554 | 0.384602 | 23 | 11 | 29 | 1.33 |
| 0.38857 | 0.238209 | 0.419714 | 0.313863 | 0.508166 | 26 | 8 | 34 | 3.33 |
| 0.45395 | 0.254911 | 0.415024 | 0.275109 | 0.481557 | 26 | 9 | 21 | 1.50 |
| 0.500837 | 0.266367 | 0.475384 | 0.318829 | 0.457728 | 24 | 11 | 30 | 0.47 |
| 0.427635 | 0.229991 | 0.379655 | 0.373993 | 0.376143 | 31 | 12 | 27 | 12.17 |
| 0.469831 | 0.261366 | 0.371044 | 0.403905 | 0.511023 | 27 | 11 | 27 | 6.00 |
| 0.408593 | 0.236678 | 0.373747 | 0.322041 | 0.411676 | 34 | 12 | 36 | 1.40 |
| 0.541703 | 0.228028 | 0.340904 | 0.362533 | 0.456058 | 22 | 9 | 28 | 2.33 |
| 0.388393 | 0.255387 | 0.320845 | 0.360574 | 0.401891 | 21 | 8 | 37 | 12.67 |
| 0.405504 | 0.255441 | 0.431642 | 0.337624 | 0.407276 | 31 | 13 | 37 | 5.33 |
| 0.492532 | 0.241437 | 0.445596 | 0.33237 | 0.420695 | 24 | 8 | 20 | 2.33 |
| 0.494087 | 0.252925 | 0.426978 | 0.360553 | 0.475124 | 36 | 9 | 29 | 2.33 |
| 0.445751 | 0.226251 | 0.411433 | 0.366573 | 0.393421 | 33 | 8 | 30 | 36.50 |
| 0.459199 | 0.261254 | 0.485573 | 0.432485 | 0.560031 | 26 | 10 | 35 | 3.33 |
| 0.46235 | 0.263443 | 0.424645 | 0.323883 | 0.411441 | 20 | 10 | 11 | 6.00 |
| 0.435409 | 0.200615 | 0.419938 | 0.38665 | 0.397042 | 45 | 10 | 25 | 11.00 |
| 0.468885 | 0.235192 | 0.372547 | 0.358564 | 0.494585 | 26 | 9 | 21 | 1.40 |
| 0.482572 | 0.270671 | 0.390871 | 0.356365 | 0.564897 | 35 | 8 | 26 | 5.00 |
| 0.508755 | 0.292086 | 0.402255 | 0.409562 | 0.443077 | 30 | 8 | 21 | 0.47 |
| 0.465654 | 0.266901 | 0.481925 | 0.371899 | 0.556801 | 32 | 8 | 27 | 6.07 |
| 0.476241 | 0.216761 | 0.417074 | 0.381165 | 0.452478 | 32 | 8 | 24 | 3.03 |
| 0.400004 | 0.221084 | 0.34872 | 0.434045 | 0.41967 | 40 | 10 | 40 | 3.33 |
| 0.398639 | 0.14657 | 0.429411 | 0.365601 | 0.318902 | 32 | 10 | 30 | 0.50 |
| 0.519844 | 0.293935 | 0.40743 | 0.271625 | 0.430805 | 18 | 7 | 17 | 6.00 |
| 0.31154 | 0.23773 | 0.383093 | 0.322795 | 0.296325 | 26 | 7 | 32 | 6.33 |
| 0.491474 | 0.244048 | 0.399979 | 0.302199 | 0.395622 | 20 | 7 | 13 | 4.33 |
| 0.414709 | 0.294962 | 0.402005 | 0.322889 | 0.481043 | 31 | 7 | 37 | 6.33 |
| 0.463023 | 0.28719 | 0.429239 | 0.373898 | 0.435959 | 21 | 7 | 20 | 12.17 |
| 0.490546 | 0.271969 | 0.463808 | 0.426576 | 0.521701 | 28 | 7 | 38 | 4.33 |
| 0.43866 | 0.29496 | 0.498192 | 0.339869 | 0.523555 | 28 | 7 | 28 | 6.00 |
| 0.423571 | 0.239126 | 0.359235 | 0.352157 | 0.49023 | 27 | 7 | 37 | 3.33 |
| 0.454069 | 0.210974 | 0.390164 | 0.401036 | 0.332898 | 24 | 7 | 20 | 2.00 |
| 0.588742 | 0.25546 | 0.432215 | 0.256568 | 0.365084 | 22 | 7 | 20 | 0.93 |
| 0.493454 | 0.214246 | 0.390133 | 0.361165 | 0.450478 | 20 | 7 | 29 | 4.00 |
| 0.425857 | 0.238578 | 0.417535 | 0.328022 | 0.397022 | 20 | 7 | 24 | 6.33 |
| 0.387155 | 0.215596 | 0.424889 | 0.400246 | 0.385028 | 28 | 7 | 33 | 0.77 |
| 0.354871 | 0.239091 | 0.413747 | 0.482721 | 0.477588 | 20 | 7 | 12 | 2.33 |
| 0.540845 | 0.295416 | 0.393855 | 0.388043 | 0.432781 | 21 | 7 | 30 | 6.33 |
| 0.449126 | 0.259301 | 0.344408 | 0.369904 | 0.429856 | 31 | 7 | 22 | 2.00 |
| 0.354336 | 0.227781 | 0.423348 | 0.389729 | 0.500503 | 30 | 7 | 23 | 0.47 |
| 0.474408 | 0.239108 | 0.355862 | 0.386457 | 0.333967 | 27 | 7 | 14 | 6.00 |
| 0.472738 | 0.286681 | 0.390632 | 0.367345 | 0.452234 | 34 | 7 | 20 | 12.00 |
| 0.570637 | 0.244506 | 0.446509 | 0.448217 | 0.468948 | 28 | 7 | 24 | 1.20 |
| 0.500674 | 0.268457 | 0.387014 | 0.359711 | 0.404956 | 32 | 7 | 29 | 7.10 |
| 0.503279 | 0.25847 | 0.446824 | 0.346268 | 0.4812 | 32 | 7 | 26 | 12.17 |
| 0.487086 | 0.291106 | 0.433499 | 0.410518 | 0.415994 | 32 | 7 | 18 | 12.17 |

Abbreviations: HAMA, Hamilton Anxiety Scale; HAMD, Hamilton Depression Scale; IFG, inferior frontal gyrus; L, left; MDD+A, anxious depression; OFG, orbital frontal gyrus; PCG, postcentral gyrus; R, right.

**Table 9**.Clinical information and mean gray matter volumes of identified clusters in each MDD-A patient

| R IFG | R OFG | Bilateral Culmen | L PCG | L Cuneus | HAMD | Anxiety scores | HAMA | Illness duration |
| --- | --- | --- | --- | --- | --- | --- | --- | --- |
| 0.379495 | 0.262177 | 0.309063 | 0.426357 | 0.360914 | 19 | 5 | 11 | 4.00 |
| 0.478418 | 0.297424 | 0.387672 | 0.277727 | 0.454868 | 19 | 6 | 27 | 4.00 |
| 0.450877 | 0.247094 | 0.324157 | 0.247822 | 0.35821 | 20 | 3 | 14 | 6.33 |
| 0.451527 | 0.209366 | 0.404597 | 0.24504 | 0.385912 | 18 | 3 | 15 | 1.33 |
| 0.475581 | 0.261782 | 0.425783 | 0.340181 | 0.473123 | 23 | 6 | 12 | 2.33 |
| 0.445231 | 0.359377 | 0.420039 | 0.295296 | 0.545525 | 19 | 6 | 22 | 6.00 |
| 0.508262 | 0.309628 | 0.448262 | 0.447683 | 0.51312 | 29 | 5 | 25 | 2.33 |
| 0.397941 | 0.231366 | 0.368705 | 0.419143 | 0.41609 | 18 | 5 | 12 | 4.00 |
| 0.440928 | 0.248919 | 0.359444 | 0.273367 | 0.406745 | 20 | 3 | 14 | 12.17 |
| 0.514121 | 0.316347 | 0.406048 | 0.330904 | 0.518468 | 20 | 6 | 15 | 6.33 |
| 0.439951 | 0.22532 | 0.377687 | 0.37026 | 0.315951 | 18 | 6 | 30 | 8.40 |
| 0.62009 | 0.271025 | 0.454895 | 0.325464 | 0.460555 | 25 | 4 | 19 | 4.00 |
| 0.445974 | 0.220284 | 0.354092 | 0.340565 | 0.339958 | 20 | 6 | 10 | 3.00 |
| 0.562575 | 0.264266 | 0.369658 | 0.304668 | 0.299223 | 19 | 5 | 16 | 6.33 |
| 0.343413 | 0.209974 | 0.320538 | 0.327003 | 0.346677 | 24 | 5 | 21 | 3.33 |
| 0.452071 | 0.201052 | 0.407667 | 0.201617 | 0.357106 | 19 | 6 | 12 | 1.00 |
| 0.468232 | 0.273069 | 0.446232 | 0.27493 | 0.373063 | 20 | 5 | 13 | 15.00 |
| 0.470414 | 0.255584 | 0.471803 | 0.21663 | 0.450384 | 17 | 5 | 22 | 36.67 |
| 0.472905 | 0.235521 | 0.41231 | 0.249448 | 0.435124 | 25 | 5 | 10 | 0.77 |
| 0.578621 | 0.265514 | 0.444537 | 0.270797 | 0.484857 | 20 | 6 | 18 | 4.00 |
| 0.506777 | 0.288098 | 0.453349 | 0.301098 | 0.618876 | 16 | 1 | 19 | 1.00 |
| 0.516098 | 0.255048 | 0.341204 | 0.42639 | 0.37132 | 20 | 4 | 13 | 25.00 |
| 0.451821 | 0.247272 | 0.417187 | 0.456088 | 0.482115 | 29 | 6 | 19 | 3.30 |
| 0.465277 | 0.269018 | 0.400634 | 0.336661 | 0.448536 | 19 | 6 | 26 | 12.67 |
| 0.394439 | 0.245198 | 0.372214 | 0.435599 | 0.484237 | 21 | 5 | 20 | 3.33 |
| 0.489784 | 0.212345 | 0.332632 | 0.367467 | 0.371587 | 28 | 6 | 25 | 12.67 |
| 0.453934 | 0.253799 | 0.487227 | 0.434351 | 0.472046 | 17 | 3 | 16 | 2.87 |
| 0.450851 | 0.220766 | 0.36564 | 0.236276 | 0.350492 | 21 | 3 | 30 | 1.00 |
| 0.517772 | 0.255133 | 0.395453 | 0.354307 | 0.36655 | 21 | 3 | 19 | 1.00 |
| 0.667194 | 0.32555 | 0.475789 | 0.394227 | 0.580291 | 24 | 2 | 13 | 1.33 |
| 0.489346 | 0.288799 | 0.369752 | 0.360893 | 0.4636 | 30 | 6 | 16 | 18.33 |
| 0.455104 | 0.267648 | 0.43451 | 0.457927 | 0.506993 | 36 | 6 | 22 | 24.00 |
| 0.509567 | 0.293734 | 0.402024 | 0.305566 | 0.457681 | 32 | 6 | 22 | 120.00 |
| 0.374952 | 0.217625 | 0.354569 | 0.304544 | 0.431686 | 29 | 6 | 11 | 12.00 |
| 0.450304 | 0.343901 | 0.522946 | 0.352431 | 0.497006 | 28 | 5 | 13 | 6.00 |
| 0.531806 | 0.319271 | 0.462217 | 0.362196 | 0.456223 | 25 | 4 | 11 | 12.17 |
| 0.562377 | 0.297987 | 0.421055 | 0.376057 | 0.486195 | 20 | 4 | 9 | 3.97 |
| 0.467043 | 0.28373 | 0.442045 | 0.574841 | 0.410947 | 25 | 4 | 14 | 24.00 |
| 0.494229 | 0.343445 | 0.440411 | 0.505976 | 0.469933 | 27 | 3 | 19 | 2.00 |
| 0.686741 | 0.305771 | 0.462939 | 0.366321 | 0.437357 | 32 | 6 | 15 | 3.03 |
| 0.552327 | 0.285149 | 0.429743 | 0.358064 | 0.40288 | 32 | 6 | 14 | 6.20 |
| 0.471495 | 0.256123 | 0.409564 | 0.431955 | 0.445015 | 32 | 6 | 24 | 12.17 |
| 0.44253 | 0.266986 | 0.402931 | 0.33317 | 0.52506 | 32 | 5 | 16 | 6.07 |
| 0.512691 | 0.330796 | 0.513998 | 0.298756 | 0.446095 | 32 | 5 | 17 | 3.03 |
| 0.554757 | 0.245494 | 0.435661 | 0.429077 | 0.488965 | 32 | 5 | 22 | 8.10 |
| 0.446639 | 0.236956 | 0.417688 | 0.26372 | 0.32395 | 32 | 4 | 14 | 3.27 |
| 0.454328 | 0.269611 | 0.407412 | 0.475942 | 0.4028 | 32 | 4 | 16 | 12.17 |
| 0.498862 | 0.269462 | 0.444393 | 0.206867 | 0.497222 | 32 | 4 | 12 | 3.00 |
| 0.41834 | 0.232417 | 0.35847 | 0.291212 | 0.348278 | 30 | 6 | 26 | 0.47 |
| 0.3902 | 0.186954 | 0.396497 | 0.453652 | 0.393725 | 13 | 6 | 9 | 0.50 |
| 0.497558 | 0.287526 | 0.384319 | 0.449953 | 0.455387 | 20 | 6 | 16 | 0.60 |
| 0.457744 | 0.286588 | 0.391239 | 0.308855 | 0.409542 | 27 | 4 | 19 | 1.30 |
| 0.554764 | 0.319817 | 0.426359 | 0.300549 | 0.386267 | 15 | 4 | 9 | 1.27 |
| 0.603294 | 0.328821 | 0.543767 | 0.344613 | 0.60725 | 20 | 4 | 5 | 2.00 |
| 0.413714 | 0.259824 | 0.300995 | 0.22996 | 0.350613 | 24 | 1 | 20 | 0.80 |
| 0.513835 | 0.277997 | 0.441227 | 0.422761 | 0.382969 | 24 | 3 | 12 | 2.00 |
| 0.430931 | 0.204396 | 0.366761 | 0.278981 | 0.371774 | 20 | 2 | 7 | 1.00 |

Abbreviations: HAMA, Hamilton Anxiety Scale; HAMD, Hamilton Depression Scale; IFG, inferior frontal gyrus; L, left; MDD-A, non-anxious depression; OFG, orbital frontal gyrus; PCG, postcentral gyrus; R, right.
